# Interactive three‐dimensional teaching models of the female and male pelvic floor

**DOI:** 10.1002/ca.23508

**Published:** 2019-11-19

**Authors:** Yi Wu, Jill P.J.M. Hikspoors, Greet Mommen, Noshir F. Dabhoiwala, Xin Hu, Li‐Wen Tan, Shao‐Xiang Zhang, Wouter H. Lamers

**Affiliations:** ^1^ Tytgat Institute for Liver and Intestinal Research, Academic Medical Center University of Amsterdam Amsterdam The Netherlands; ^2^ Institute of Digital Medicine, College of Biomedical Engineering and Imaging Medicine Army Military Medical University Chongqing China; ^3^ Department of Anatomy & Embryology Maastricht University Medical Center Maastricht The Netherlands

**Keywords:** adiposity, levator ani muscle, anal sphincter, rectourethral muscle, perineal body, urethral sphincter, sexual dimorphism, vaginal support

## Abstract

Controversies regarding structure and function of the pelvic floor persist because of its poor accessibility and complex anatomical architecture. Most data are based on dissection. This “surgical” approach requires profound prior knowledge, because applying the scalpel precludes a “second look.” The “sectional” approach does not entail these limitations, but requires segmentation of structures and three‐dimensional reconstruction. This approach has produced several “Visible Human Projects.” We dealt with limited spatial resolution and difficult‐to‐segment structures by proceeding from clear‐cut to more fuzzy boundaries and comparing segmentation between investigators. We observed that the bicipital levator ani muscle consisted of pubovisceral and puborectal portions; that the pubovisceral muscle formed, together with rectococcygeal and rectoperineal muscles, a rectal diaphragm; that the external anal sphincter consisted of its subcutaneous portion and the puborectal muscle only; that the striated urethral sphincter had three parts, of which the middle (urethral compressor) was best developed in females and the circular lower (“membranous”) best in males; that the rectourethral muscle, an anterior extension of the rectal longitudinal smooth muscle, developed a fibrous node in its center (perineal body); that the perineal body was much better developed in females than males, so that the rectourethral subdivision into posterior rectoperineal and anterior deep perineal muscles was more obvious in females; that the superficial transverse perineal muscle attached to the fibrous septa of the ischioanal fat; and that the uterosacral ligaments and mesorectal fascia colocalized. To facilitate comprehension of the modified topography we provide interactive 3D‐PDFs that are freely available for teaching purposes. Clin. Anat. 33:275–285, 2020. © 2019 Wiley Periodicals, Inc.

## INTRODUCTION

The pelvic floor plays an important role in urinary and fecal continence and pelvic‐organ support. Despite years of study controversies regarding its structure and function do, however, persist because of its poor accessibility and complicated anatomical architecture.

### Dissectional Versus Sectional Approach to Anatomy

Most anatomical investigations of the lesser pelvis used the “dissectional” approach. The dissectional approach is that of the surgeon and requires profound prior knowledge, because structures must be identified on inspection. In line, the low‐power lenses that are used in laparoscopic dissection have led to the identification of new, surgically relevant structures (Jimenez and Aguilar, 2009). Perhaps even more important, a “second look” is impossible due to the irreversible results of applying the scalpel.

These limitations do not apply to the “sectional” approach, but this method, best known for its use in conjunction with microscopes, has limitations with respect to the size of the structure that can be studied (presently ≤45 * 15 cm). Larger specimens, such as adult human bodies, are visualized with an episcopic approach (Kathrein et al., 1996; Spitzer et al., 1996). This variant of the sectional approach visualizes the surface of the part of the specimen that remains in the tissue block after a section of 0.1–1.0 mm is “removed” with a surface grinder. Accessibility of structures is, therefore, not an issue. The necessity to reconstruct the segmented structures to obtain 3D images is also hardly a problem, because episcopic sections are in register and undeformed. This approach has resulted in several “Visible Human Projects” (Dai et al., 2012).

Three‐dimensional models of the greater and lesser pelvis of the females of the Visible Human Project (USA) and the Visible Korean Project have been published (Sergovich et al., 2010; Shin et al., 2013). Both reports focused on the technical advances in presenting complex anatomical configurations. The components of the Korean model in particular can be inspected smoothly via an interactive 3D‐PDF. Unfortunately, neither the American nor the Korean study addressed the clinical anatomy of the pelvic floor in detail and did not report whether their findings shed new light on pelvic anatomy.

We recently studied the pelvic floor of the Chinese Visible Human and presented our findings as interactive 3D‐PDFs of almost 50 structures (Wu et al., 2015; Wu et al., 2017; Wu et al., 2018). The main challenges of the “Visible Human” approach are the limited spatial resolution in the “section” and the dependence of segmentation on differences in structure and color that are present in the tissue block before sectioning. We dealt with these hurdles by gradually filling in the overall picture: we always labeled the easy‐to‐segment (parts of) structures first and then proceeded to the more‐difficult‐to‐segment structures. Furthermore, we re‐evaluated the outcome several times by reinspecting the sections and, in complex cases, by comparing the segmented images prepared separately by different operators.

The sectional approach as delineated earlier has produced several new findings with respect to the topographic anatomy of the pelvic floor, which we consider relevant for teaching pelvic anatomy and which are incorporated in the published 3D models. The present brief review summarizes these new findings. However, due to the irregular boundaries that result from manual segmentation of the contours of structures or organs, it is difficult for students to get a feel for the position and mutual topographic relations of the reconstructed features. To facilitate comprehension we have, therefore, applied remodeling software that allows accurate smoothing of the surface of reconstructed structures and organs. These new interactive 3D‐models are freely available for use in the classroom.

## MATERIAL AND METHODS

Consecutive sections of five female (CVH2, −4, −5, CVO and VHF (VHP female)) and five male specimens (CVH1, −3, VHM (VHP male), and two 26‐week fetuses (Leiden S2289 and S2600)) were studied in detail (Wu et al., 2015; Wu et al., 2017; Wu et al., 2018). We focused on the pelvic floor. All identified structures in the CVH1 male and the CVH5 female specimens were used to assemble a detailed 3D reconstruction using the Amira software package (http://www.amiravis.com). Furthermore, partial reconstructions of the other specimens were prepared to confirm that the features we highlight in CVH1 and CVH5 represented a typical anatomical configuration. The original photographs of CVH5 (transversely sectioned) and CVO (sagittally sectioned) can be found in Wu et al. (2017)). Polygon meshes from all reconstructed materials were exported via “vrml export” to the remodeling software Cinema 4D (MAXON Computer GmbH, Friedrichsdorf, Germany). The accuracy of the remodeling process was safeguarded by simultaneous visualization in Cinema 4D of the original output from Amira and the remodeled Cinema model (Supporting Information Fig. S1). Subsequently, the Cinema 3D‐model was exported via “wrl export” to Adobe portable device format (PDF) reader version 9 (http://www.adobe.com) for the generation of 3D‐interactive PDF files that are provided online (Supporting Information Figs. S2 and S3). Whereas we mostly refer in the text to the figures, the reader is encouraged to simultaneously inspect the interactive PDFs, because their rotational options (“live” images) allows a much better understanding of the complex local topography than the “still” pictures in the images. In our description, we use superior, inferior, anterior, and posterior for the description of topographical relations.

## NEW FINDINGS

The study of five male and five female sectioned specimens modified our views on the anatomy of the levator ani muscle (LAM), anal sphincter, perineal body, urethral sphincter, mesorectum, and vaginal supports.

### Levator Ani Muscle Complex

#### *Sexual dimorphism*

**Figure 1:**
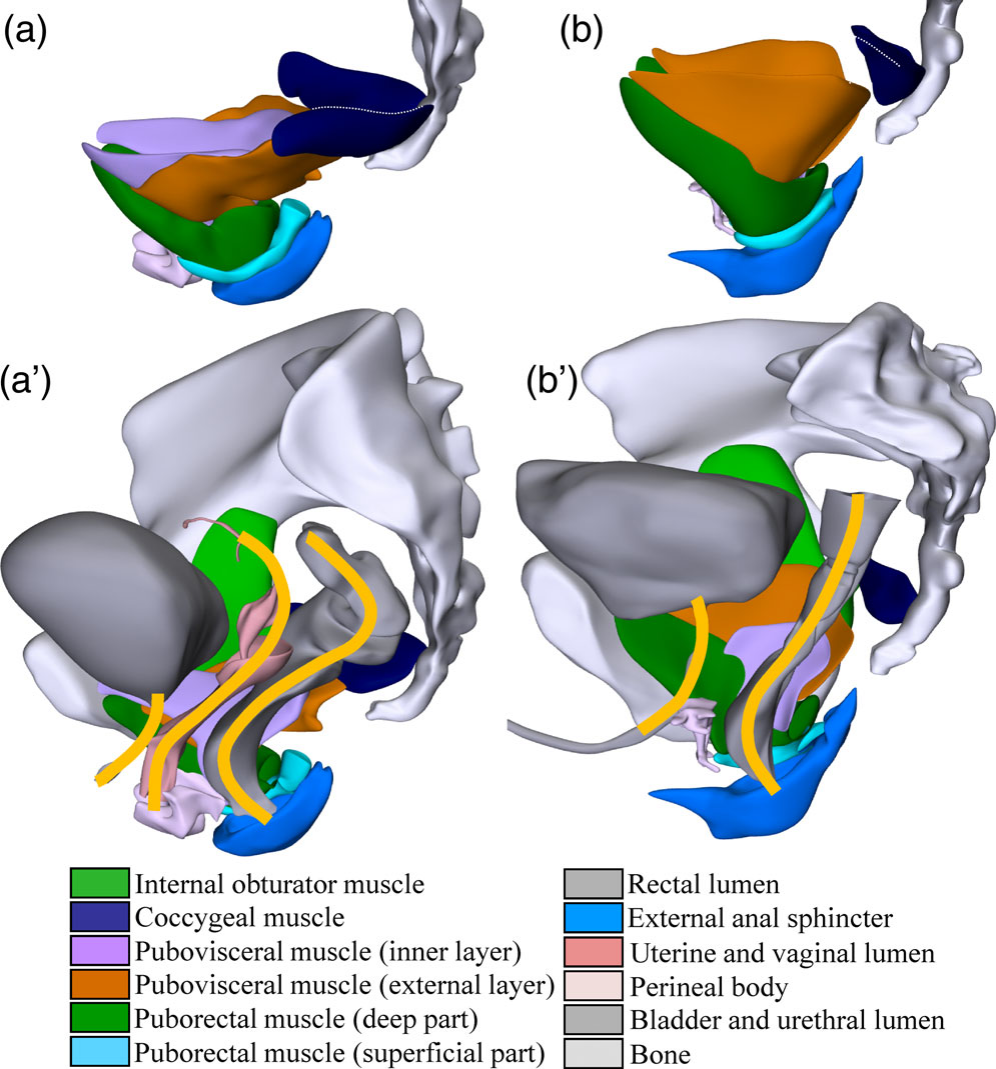
Comparison of the female (left) and male (right) pelvic floor. Left lateral views. Top row emphasizes contrast between the shallow and wide female (**a**), and the steep and narrow the male pelvic floor (**b**). Further note the elongated shape of the EAS in males relative to females, with long anterior and posterior spurs. The bottom row shows the course of the center of the lumen of the pelvic organs projected unto the right muscles of the pelvic floor in females (**a′**) and males (**b′**). Note the inverted curvature of rectal and urethral axes, the more pronounced bending of the rectum and anus in females than in males, and the more pronounced bending of the rectal compared to the uterovaginal lumen. Structures are identifiable by their color code (see also Supporting Information supplemental Fig. S4). [Color figure can be viewed at http://wileyonlinelibrary.com]

In agreement with present‐day images of the LAM, our studies found the muscle to be funnel‐shaped, with attachments to the pubic bone anteriorly, and the internal obturator muscle and ischial spine laterally (Fig. 1A,B). The fibrous attachment of the LAM to the internal obturator muscle (its “tendinous” arch) was remarkably inconspicuous. Posterosuperiorly, the LAM bordered the largely fibrous coccygeal muscle. The LAM is better developed in females than males, which is remarkable because the overall muscle mass of females is only 70–75% of that of males (Wang et al., 2001; Abe et al., 2003). We have attributed the difference to the more transverse orientation and, hence, less favorable leverage of the muscles in females than males. An important consequence of the shallower LAM funnel in females than in males is the more pronounced “S”‐shaped configuration of the distal rectum and anal canal. The posterior part of the LAM, therefore, not only supports the distal rectum, but also the uterovaginal junction in a roof‐tile fashion (Berglas and Rubin, 1953; Béthoux and Bory, 1962) (Fig. 1A′,B′).

#### *Puborectal muscle is part of anal sphincter complex*

**Figure 2:**
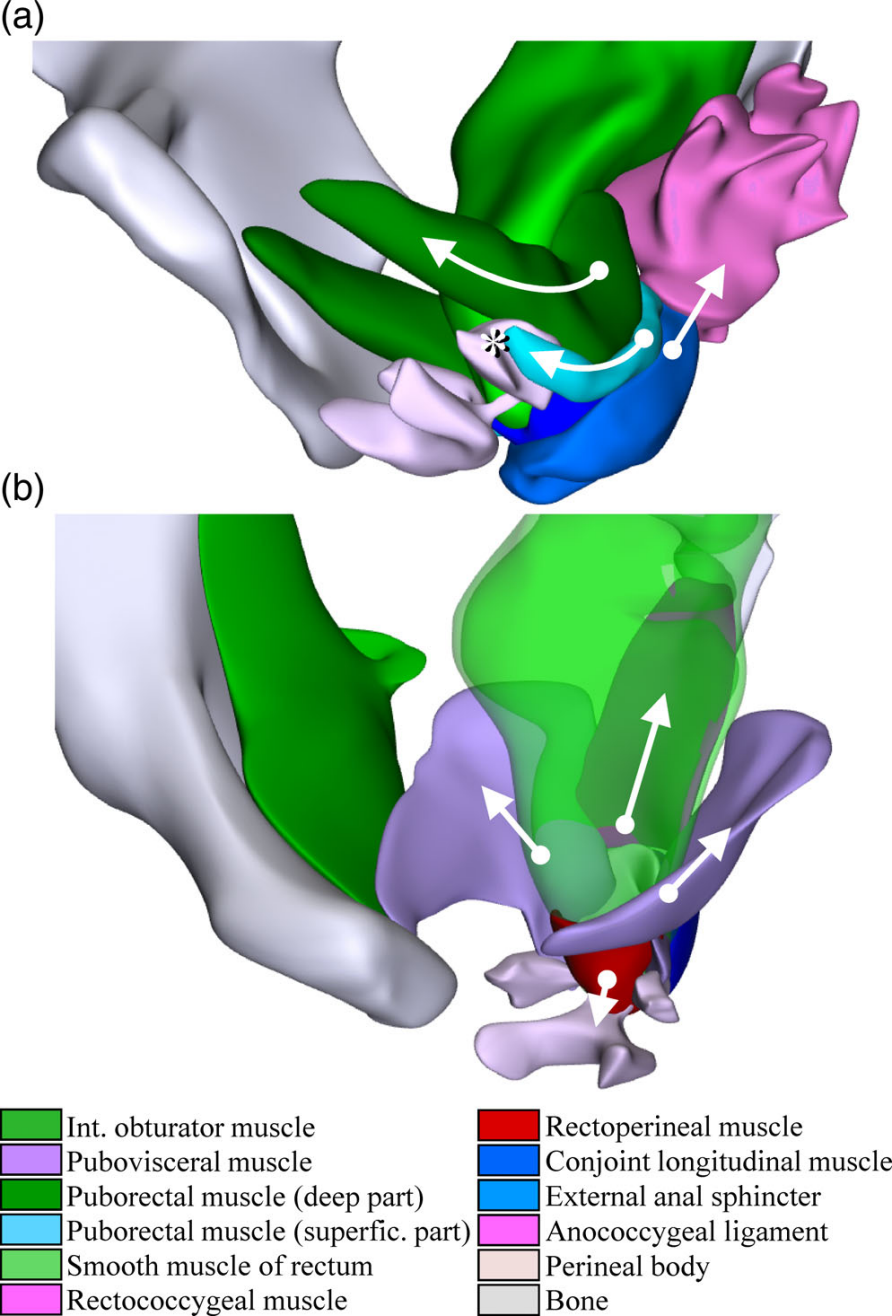
The sphincters of the hindgut. (**a**) Relation between the puborectal portions of the levator ani muscle and the external anal sphincter. Note attachments (*) of superficial and deep puborectal muscles on the deep part of the perineal body. Further note that anococcygeal ligament connects external anal sphincter with coccygeal bone. Arrows indicate movement upon contraction. (**b**) Configuration of the rectal diaphragm. The rectal diaphragm is attached to the pelvic fascia laterally, the coccygeal bone posteriorly, and the perineal body anteriorly. Arrows indicate direction of force upon contraction. Structures are identifiable by their color code (see also Supporting Information Fig. S4). [Color figure can be viewed at http://wileyonlinelibrary.com]

Although the anterior portion of the LAM was well developed, there were no perimysial septa at this location that allowed a subdivision into puborectal, pubococcygeal, and iliococcygeal portions, as shown in virtually all anatomical textbooks. More posteriorly, however, fibrous septa and the orientation of muscle fibers allowed separation into the puborectal muscle inferiorly and the “pubovisceral” muscle superiorly. The topography of the puborectal muscle, with a muscle sling that passed the anorectal bend posteriorly, was similar to that depicted in anatomical textbooks. Unlike these textbooks, but in agreement with Fritsch and colleagues, who also used the sectional approach (3–5 mm‐thick epoxy‐resin sections; (Fritsch et al., 2002)), we found that the puborectal muscle colocalized with the “deep portion” of the external anal sphincter (EAS). Both names, therefore, represent one‐and‐the‐same muscle (Table 1). Superficial and deep portions of the puborectal muscle, which correspond to superficial and deep portions of the anal sphincter, could be identified posteriorly where the puborectal muscle passed behind the anorectal junction. Anteriorly, the superficial portion of the puborectal muscle/anal sphincter attached to the perineal body, whereas the deep portion attached, in addition, to the pubic bone (Fig. 2A). While we retained two components in the puborectal muscle (Table 1), Fritsch and colleagues (Fritsch et al., 2002) considered the puborectal a single muscle.

**Table 1:** Terminology of Pelvic‐Floor Muscles in Present Study Compared to that in Anatomical Terminology (Terminol, 1998)

Present study	Anatomical Terminology (TA)
Levator ani (bicipital)	Puborectal **+** (pubococcygeal **+** iliococcygeal)
Pubovisceral	Pubococcygeal **+** iliococcygeal
Puborectal (deep portion)	Puborectal **≡** deep portion of EAS
Puborectal (superficial portion)	Superficial portion of EAS
External anal sphincter proper	Subcutaneous portion of EAS

#### *Pubovisceral versus pubo‐ and ilio‐coccygeal muscles*

We were unable to identify the pubo‐ and ilio‐coccygeal muscles as adjacent but separate parts of the LAM, as shown in anatomical textbooks. Instead, we identified a configuration that we labeled “pubovisceral,” because this part of the LAM inserts into the wall of the anorectum (Table 1). The term “pubovisceral” was proposed by DeLancey *c.s*. because of its insertion on the pelvic organs (Kearney et al., 2004) but, in agreement with Fritsch and colleagues (Fritsch et al., 2002), we could not demonstrate attachment to the vagina (Wu et al., 2015). The pubovisceral muscle had inner and outer muscle layers: its inner layer attached to the perineal body and the conjoint longitudinal muscle of the rectum inferiorly, and to the fascia surrounding of the rectococcygeal muscle posteriorly, while its outer layer consisted of patchy muscle sheets that only partially overlapped with the inner layer and also attached to the rectococcygeal fascia posteriorly. Fritsch and colleagues deduced an intermediate configuration, with a substantial overlap of the superomedial pubococcygeal and inferolateral iliococcygeal muscles (Frohlich et al., 1997). The puborectal and pubovisceral portions of the LAM shared their anterior insertion on the pubic bone, but differed in their posteroinferior course, so that the LAM can be considered a bicipital muscle (Fig. 1A,B). Muscle fiber orientation in the anterior portion of the LAM inclined ~30° and in the puborectal and pubovisceral muscles lateral to the rectum ~45° relative to the transverse plane, while the orientation in the superior portion of the pubovisceral muscle was predominantly transverse. These numbers correspond fairly well with those estimated on MRIs (Betschart et al., 2014).

#### *Rectococcygeal muscle*

The smooth (“unstriped” (Smith, 1908)) rectococcygeal muscle is also described as the “hiatal ligament” (Shafik, 1999), “anterior layer of the anococcygeal ligament” (Kinugasa et al., 2011), “thick smooth muscle on the surface of the levator ani muscle” (Tsukada et al., 2016), or “posterior midline raphe of the LAM” (Stein and DeLancey, 2008). The transformation of smooth muscles from a contractile to a synthetic (fibrous) phenotype during one's lifetime is often seen (Beamish et al., 2010), but in the young specimens that we studied (Wu et al., 2015), the structure had all features of a well‐delineated (smooth) muscle.

#### *Rectoperineal muscle*

The longitudinal smooth‐muscle layer of the rectum continued distally into the conjoint longitudinal muscle of the rectum. The latter derives its name from anterolateral contributions of the inner layer of the pubovisceral muscle to the muscle. Accordingly, the muscle is described to contain smooth muscle internally and striated muscle fibers externally (Macchi et al., 2008; Kim et al., 2015; Muro et al., 2019), with the striated component possibly declining with age. The conjoint muscle reached up to the anorectal junction posteriorly and laterally, but anteriorly, it could only be identified inferiorly, because a few thick muscle bundles of the longitudinal smooth muscle of the rectum did not follow the anorectal bend, but continued downward to the perineal body. These smooth muscle bundles were described as “rectovaginal” or “rectourethral,” or independent of the sex, “rectoperineal” muscle (Brooks et al., 2002; Sebe et al., 2005; Zhai et al., 2011). The rectoperineal muscle was widest superiorly and tapers off toward its insertion on the perineal body. This muscle was very well developed in VHM, an acknowledged body builder, suggesting it supports the pelvic floor.

#### *Rectal diaphragm*

Inspection of Supporting Information Figures S2 and S3 shows that the anal canal was suspended at the anorectal junction by strands of the longitudinal smooth muscle of the rectum to the coccygeal bone posteriorly (rectococcygeal muscle) and to the perineal body anteriorly (rectoperineal muscle). Laterally, the anorectal junction was suspended by the inner layer of both pubovisceral muscles via their continuity with the conjoint longitudinal muscle of the rectum (Fig. 2B). This rectal “diaphragm,” which was oriented perpendicularly to the axis of the anal canal, has not yet been recognized to our knowledge. It forms the inferior boundary of the mesorectum and is, therefore, an important surgical structure. Functionally, we hypothesize that it is involved in defecation, because its contraction lifts the anorectal junction.

### Anal Sphincter Complex

According to most anatomical textbooks, the EAS consists of subcutaneous, superficial, and deep parts. As described earlier, the superficial and deep parts of the EAS corresponded with the superficial and deep portions of the puborectal muscle, respectively (Table 1). The well‐developed subcutaneous portion of the EAS enveloped the rectum and internal anal sphincter completely, thus forming the only “real” sphincter. Hence, we denoted this portion the EAS “proper.” On its posterior side, the anococcygeal ligament fixed the EAS to the coccygeal bone (Fig. 2A). On its anterior side, the EAS passed the superficial transverse perineal and bulbospongious muscles superficially in the female (Wu et al., 2017), while a midline spur extended even further anteriorly in the male (Arakawa et al., 2010; Wu et al., 2018). We cannot exclude that some muscle fibers connect EAS and bulbospongious muscle, as reported recently in a diffusion tensor‐imaging study of perineal muscles (Zifan et al., 2018). However, the putative exchange area only accounted for <25% of the height of the EAS.

### Perineal Body

The perineal body was an irregular fibromuscular node in the wedge‐shaped space between the lower portion of the rectum and the vagina (female) or urethra (male). Its manifold fibrous extensions served as attachment for muscles. In agreement with earlier studies (Oh and Kark, 1973; Aigner et al., 2004; Zhai et al., 2011), we found superficial and deep portions: The deep portion served as attachment for the medial layer of the pubovisceral muscle, and the deep and superficial portions of the puborectal muscle, while the superficial portion served as attachment for the rectoperineal, deep perineal, superficial transverse perineal, and bulbospongious muscles. Our finding that its volume was ≥ twofold larger in young females than males underscores its role as a crucial component of the functional pelvic floor.

#### *Deep perineal muscle*

**Figure 3:**
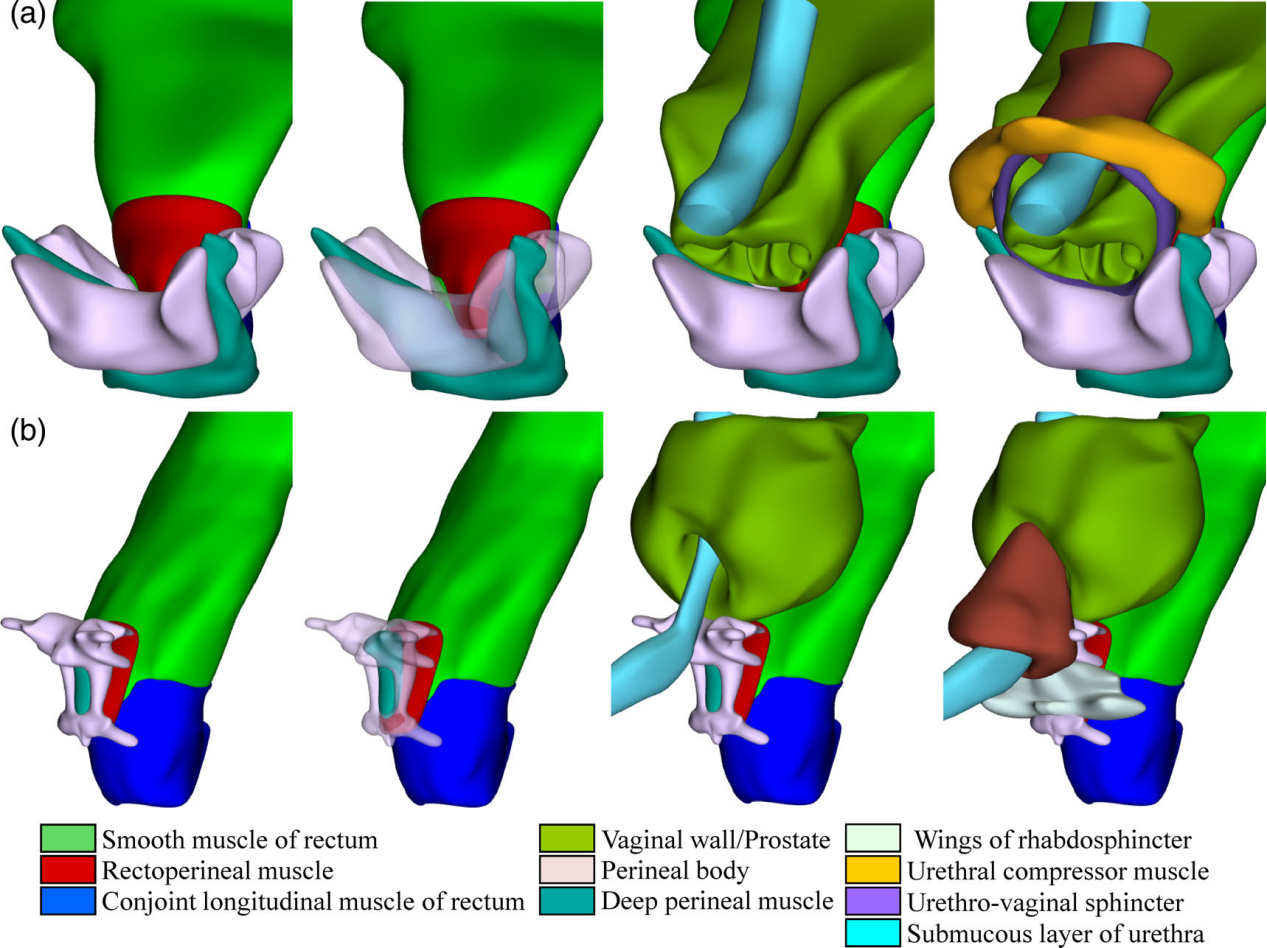
The relation between the perineal body and the rectoperineal and deep perineal muscles in females (**a**) and males (**b**). The smooth muscle wall of the rectum and its anterior continuation into the rectoperineal and deep perineal muscles is shown in the left two columns, with the perineal body rendered transparent in the second column. The topographic relation to the urethra and vagina or prostate is shown in the third column, with the components of the urethral sphincter are added in the fourth column. Structures are identifiable by their color code (see also Supporting Information Fig. S4). [Color figure can be viewed at http://wileyonlinelibrary.com]

The status of the deep (transverse) perineal muscle is contentious. This smooth muscle has variously been declared nonexistent (Dorschner et al., 1999) or identified as a “puboperineal” muscle that stretched > threefold during delivery (Lien et al., 2004). Only smooth muscles allow that degree of stretching without injury (Seow and Solway, 2011). Accordingly, several studies have confirmed its status as a smooth muscle (Nakajima et al., 2017; Muro et al., 2018, 2019). The developmental anatomy of the rectourethral muscle and perineal body can account for much of the controversies that surround the deep perineal muscle (Table 2). In fetuses a well‐developed and well‐delineated anterior slip of the longitudinal smooth muscle of the rectum extended toward the urethral rhabdosphincter as the so‐called rectourethral muscle (Wu et al., 2018). When the (superficial portion of the) perineal body began to form prenatally inside this smooth muscle as a jagged fibrous structure, the part posterior to the perineal body became the rectoperineal muscle and the part anterior to it the deep perineal muscle (Table 2). In females, the deep perineal muscle divided anteriorly into left and right wings alongside the vagina (Fig. 3A) (Wu et al., 2017). Due to the limited development of the perineal body in males lateral fibers of the rectourethral muscle continue to bypass it (Fig. 3B), so that this muscle persists into adulthood (Zhai et al., 2011). In females, by contrast, the fibrous tissue of the perineal body often extends into and (partially) transforms the deep perineal muscle into the fibrous “perineal membrane” (Oelrich, 1983; Stein and DeLancey, 2008; Brandon et al., 2009), indicating that the perineal body expands as a regenerative response to wear and tear during life. This explanation is underscored by the relative persistence of the original architecture of the deep perineal muscle in elderly Japanese males (Nakajima et al., 2017) and its much less structured appearance in elderly Japanese females (Muro et al., 2019). The age‐related expansion of fibrous tissue further explains why striated muscles like the levator ani, superficial transverse perineal, and bulbospongious muscles become secondarily attached to the perineal body.

**Table 2:** Arrangement of Rectourethral Muscle in Males and Females

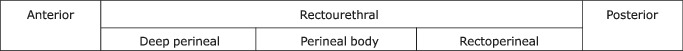

For details, see main text.

Although the perineal body is an acknowledged item in “Anatomical Terminology” (A09.5.00.0005 (Terminol, 1998)), it is not universally accepted as a specific structure. Thus, Akita and colleagues argue that description of the perineal body as a node is a too delimited description and should, instead, be considered a region (Muro et al., 2018). However, our description of the perineal body as a fibromuscular structure in the wedge‐shaped space between the lower portion of the rectum and the vagina or urethra (“region”) that penetrates with many antenna‐like extensions between the fibers of the rectourethral muscle (“shape”) (Wu et al., 2015; Wu et al., 2017; Wu et al., 2018) is not very different from the impression that emanates from their trichrome‐stained illustrations of the rectourethral muscle (Muro et al., 2018). We, therefore, conclude that, not uniquely, both groups describe the same observation with different terminologies.

### Mesorectum

The mesorectum is the perirectal space that is filled mostly with fat, lymph nodes, but remarkably few veins, and that is delimited by the rectal adventitia. Three‐dimensionally, the mesorectum was an anteriorly concave, inverted cone. Superiorly, the mesorectum surrounded the rectum on all sides, but inferior to the rectouterine or rectovesical recess the rectum was directly apposed to the vagina or prostate, respectively. The common part of the adventitia of rectum and vagina or prostate is known as “Denonvilliers’ fascia.” Here again, it is useful to recall its developmental anatomy to understand the adult configuration. In the embryo, the coelomic cavity extends down to the muscles of the pelvic floor, as it still does in all adult quadrupeds. In humans, however, this downward extension of the peritoneal cavity disappears in much the same way as the ascending and descending portions of the colon become retroperitoneal (Hikspoors et al., 2019). Denonvilliers’ fascia has, therefore, the same origin as Toldt's fascia in the abdomen (Toldt, 1893; Tobin and Benjamin, 1945). Although the coelomic space between rectum and vagina or prostate disappears in the seventh week of development (Tobin and Benjamin, 1945), Denonvilliers’ fascia reportedly develops only after birth (Kraima et al., 2015), again probably resulting from wear and tear due to the movements of rectum and vagina or prostate. The discussion of whether Denonvilliers’ fascia consists of one or two layers is, therefore, futile.

### Urethral Sphincter Complex

#### *Female sphincter*

**Figure 4:**
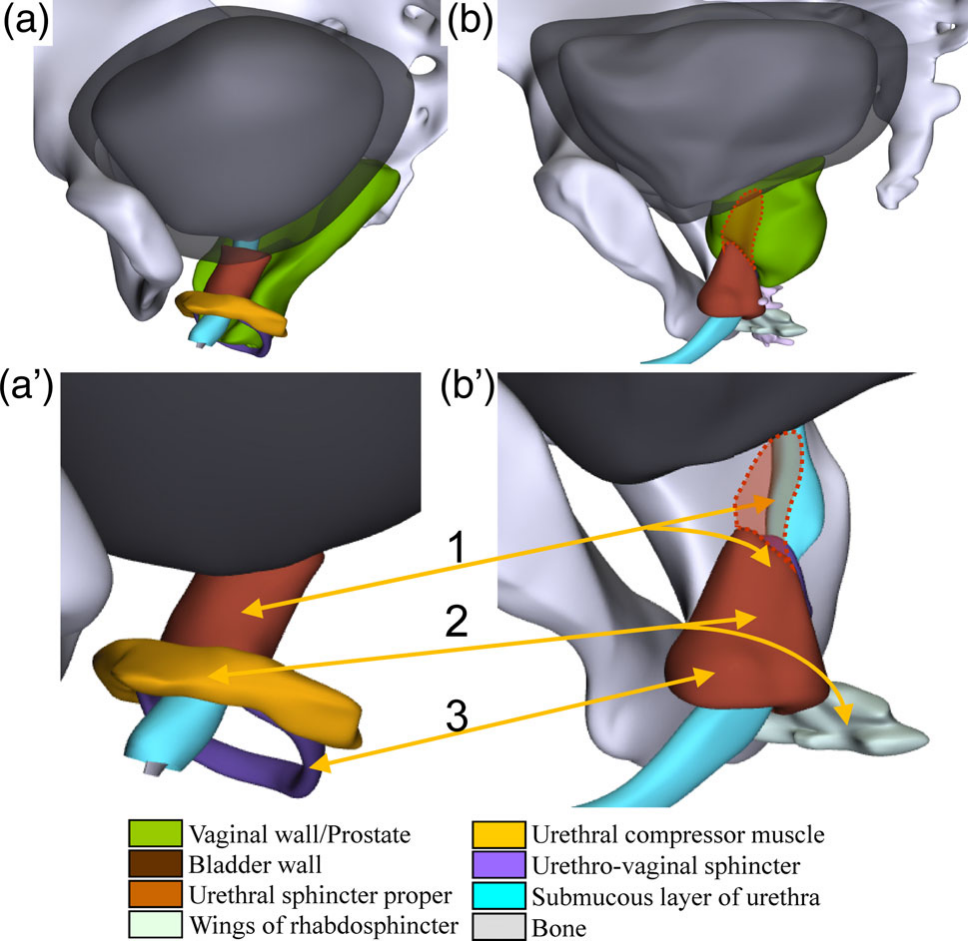
The striated sphincter of the urethra in females and males. Top panels show position of the sphincter relative to urethra and vagina (**a**) or prostate (**b**), while the bottom panels indicate the three homologous components of female (**a′**) and male (**b′**) sphincter by arrows. Please note that the detectable extension of the urethral sphincter over the anterior portion of the prostate was small in CVH1 and was found as a thin layer extending to the bladder neck in histologically processed sections (transparent areas). Both female and male urethral sphincters extend from the bladder neck along the pelvic part of the urethra. Its middle part (2) is best developed in females (urethral compressor), whereas its inferior part (3) is best developed in males (membranous portion of urethral sphincter). Structures are identifiable by their color code (see also Supporting Information Fig. S4). [Color figure can be viewed at http://wileyonlinelibrary.com]

The adult female urethra was ~4 cm long, of which the striated urethral sphincter complex covered only the upper ~80%. In agreement with earlier histological studies (Oelrich, 1983), we distinguished three components: the urethral sphincter proper superiorly, the urethral compressor, and the urethrovaginal sphincter inferiorly (Fig. 4A). The sphincter proper encircled the upper portion of the urethra and contained a tendinous raphe in the posterior midline, which does not affect its function as a sphincter. The U‐shaped compressor muscle surrounded the urethra anteriorly and laterally at the transition of the upper two‐third into the lower one‐third. Posteriorly, the compressor muscle passed the vaginal wall to insert into the fascia of the deep puborectal muscle, just anterior to the attachment of that muscle to the perineal body. The urethral compressor muscle did not attach on the pubic bone, as usually shown (Oelrich, 1983). This configuration was already seen in the fetus (Wallner et al., 2009) and implies that an impaired function of the puborectal muscle due to, for example, a laceration affects the function of this part of the urethral sphincter. The poorly developed urethrovaginal sphincter surrounded the urethra and the distal vagina. Pressure profiles of the urethra show that the compressor is the most powerful part of the sphincter complex (Constantinou, 2009).

#### *Male sphincter*

The male striated urethral sphincter complex consisted of a circular portion around the membranous urethra between the penile bulb and bulbospongious muscle inferiorly and the base of the prostate superiorly (Oelrich, 1980; Kaye et al., 1997; Gil‐Vernet et al., 2016; Wu et al., 2018). The prostatic part of the rhabdosphincter was semicircular and extended upward on the anterior half of the prostate toward the base of the bladder as a thinning and narrowing muscle sheet (Fig. 4B). The male rhabdosphincter had two small posteroinferior wings that passed the rectourethral muscle laterally (Zhai et al., 2011; Wu et al., 2018).

#### *Sexual dimorphism*

The architectural plan of the rhabdosphincter is similar in both sexes: Inferiorly, the sphincter is circular, whereas it only covers the anterior and lateral portions of the urethra more superiorly (Fig. 4A′, B′). The presence or absence of the prostate determines the appearance of upper portion of the sphincter (absence or presence, respectively, of a posterior raphe). The transition between the inferior circular and superior U‐shaped portion of the urethral sphincter is just inferior to the entrance of the deferent ducts into the urethra in the male and that of the vagina into the vestibule in the female. These positions correspond to the adjacent entrances of the Wolffian and Müllerian ducts into the embryonic urogenital sinus. Sexual dimorphism results from differential development of the respective portions of the sphincter (Fig. 4A′,B′): the inferior, circular “membranous” portion is best developed in males, whereas the middle, U‐shaped compressor portion is best developed in females (Wu et al., 2018). The wings of the male sphincter appear to represent the lateral attachments of the compressor muscle in the female, again with a clear difference in development between both sexes.

### Vagina and Its Supports

#### *Vaginal shape*

The vagina is often depicted as a tubular structure. In agreement with a few other studies (Pendergrass et al., 2000), our observations showed that this representation is incorrect (Wu et al., 2017). The outer shape of the vagina near its junction with the vestibule was circular on cross‐section, while its lumen resembled that of a purse‐string suture. At this position, we identified the urethrovaginal sphincter on its lateral and posterior surface. More superiorly, the cross‐sectional shape of the vagina became trapezoid, with the vaginal lumen resembling the letter “H.” Here, the anterior and posterior vaginal walls covered the urethra and rectoperineal muscle, respectively. At the level of the bladder neck and anorectal bend, the folds in the vaginal wall flattened and the lumen widened. Despite these marked differences in shape the perimeter of the vaginal lumen was similar along the entire vagina. At its superior end, the vaginal fornix forms an inverted cone around the cervix. The distance from the vaginal orifice to the tip of the anterior vaginal fornix is ~25% shorter than that to the posterior fornix.

#### *Vaginal fixation*

The bulbospongious muscles guarded the entrance to the vagina just below the pelvic floor and surrounded the vestibular bulbs anteriorly and Bartholin's glands posteriorly. Medial to Bartholin's gland, the urethrovaginal sphincter surrounded the vaginal introitus. Well‐developed fibrous tissue connected the vaginal wall to the urethra anteriorly, to the deep perineal muscle laterally and its continuation into the perineal body posteriorly. More superiorly, however, only loose areolar tissue containing many large veins was present between the vaginal wall and the medial layer of the pubovisceral muscle. Above the pelvic floor Denonvilliers’ fascia formed the connection between the vaginal and rectal walls. The vaginal wall was, thus, surrounded and fixed by dense connective tissue inferiorly, whereas it was only supported by loose connective tissue and a rich venous plexus above the urethral compressor and Denonvilliers’ fascia (Wu et al., 2017).

In this respect it is of interest, the uterosacral ligaments as described by gynecologists colocalize with the mesorectal fascia as described by radiologists and surgeons (e.g., (Fritsch and Hotzinger, 1995; Umek et al., 2004)). The absence of any histological evidence for fibrous pelvic‐organ support near the cervicovaginal junction in at least 10 studies over the last 90 years (Wu et al., 2017) suggests that, rather than a few strong suspending ligaments, the cooperation of many “weak forces” is necessary to retain the pelvic organs at their normal position (Cosson et al., 2013). These weak forces impress as “ligaments” when strands of tissue between the pelvic organs and the lateral pelvic wall, such as the sacrouterine and perhaps the cardinal ligaments, tighten upon extension, but fail to suspend these organs in the long run when used to repair a prolapse. In this respect, these “ligaments” have been likened to chicken wire or a woven net: they tighten locally when pulled apart, but fail when loaded permanently (Range and Woodburne, 1964; De Caro et al., 1998).

**Figure 5:**
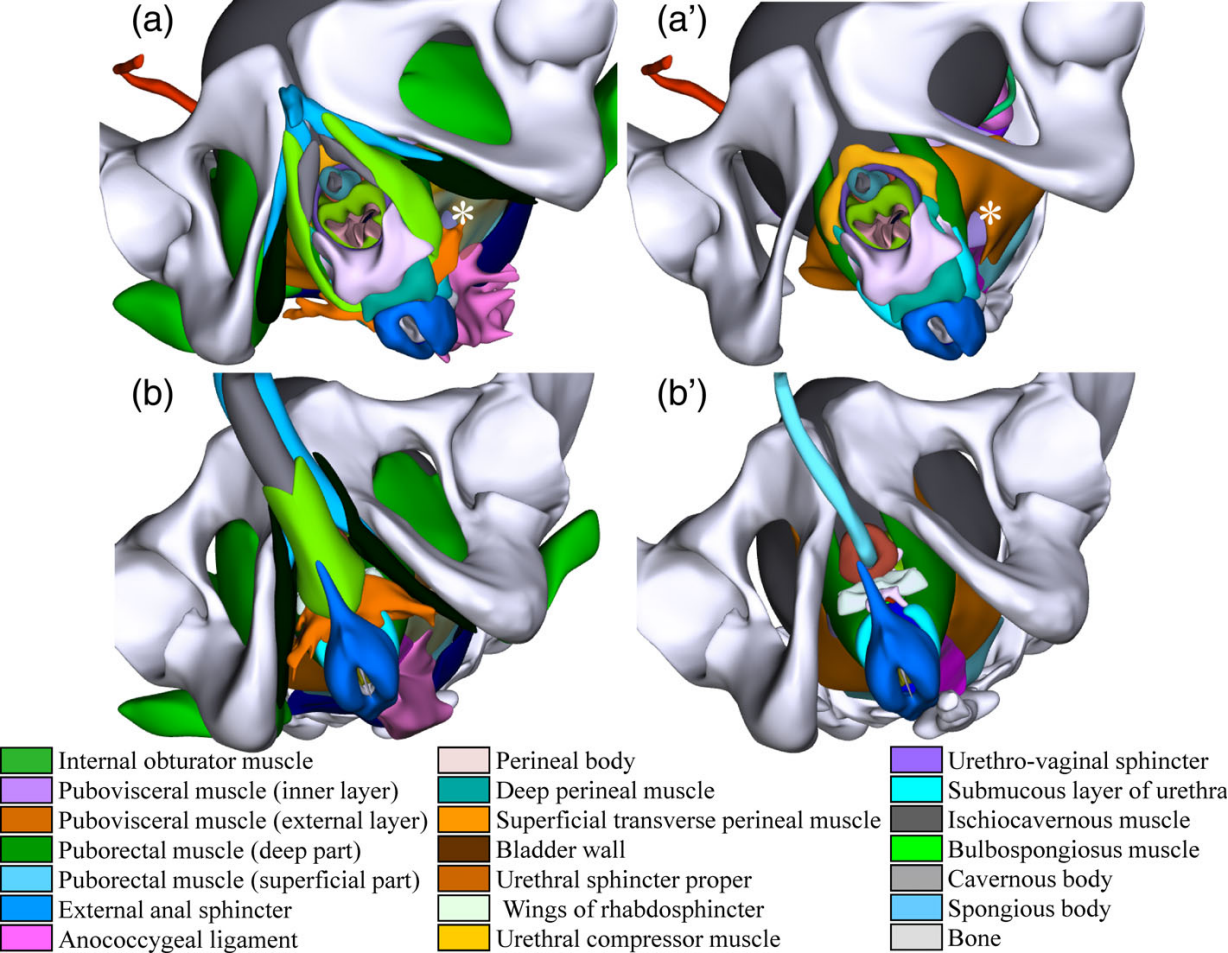
The pelvic floor in females and males. The left column shows the position of all reconstructed structures in the pelvic floor of females (**a**) and males (**b**). Note the relatively unguarded urethral and vaginal orifices, as well as the large muscular hiatus between the puborectal muscle and the ischial bone (asterisk) in the female. The right column (**a**′ and **b**′) shows the configuration of the perineal body and associated structures in females and males after removal of the ischiocavernous, bulbospongious, and superficial transverse perineal muscles. Please note small size of perineal body in the male. Structures are identifiable by their color code (see also Supporting Information Fig. S4). [Color figure can be viewed at http://wileyonlinelibrary.com]

The conclusion that fibrous tissue does not define the “suspensory ligaments of the uterus” is underscored by the finding that all structures that can contribute to the support of the pelvic organs are necessary to keep these organs in place (Cosson et al., 2013). In this respect, we already referred to the roof‐tile arrangement of anorectum and uterine cervix (Fig. 1A′). The finding that the muscles in the urogenital triangle (ischiocavernous, bulbospongious, and superficial transverse perineal) were hypertrophied in the VHM specimen, who practiced bodybuilding, indicates that these muscles contribute to pelvic floor support in the male. Comparison of the female (Fig. 5A) and male pelvic floor (Fig. 5B) shows that the entrance of the vagina and urethra into the vulva is, nevertheless, a weak spot. The elastic moduli of vagina, uterus, cardinal, sacrouterine, and round ligaments are similar, whereas that of the cervix is smaller (Chantereau et al., 2014; Baah‐Dwomoh et al., 2016). In agreement, biomechanical studies identify the fixation of vagina and cervix as most prone to fail (Brandao et al., 2016; Peng et al., 2016).

#### *Fibrous fat septa*

The superficial transverse perineal muscle did not to attach near the ischial tuberosity, as shown in anatomical textbooks, but on well‐developed fibrous septa in the fat of the ischio‐anal fossa. Contraction tightens the fibrous septa and confers stability on the fat body in the ischio‐anal fossa. Although this mechanism may support the space between the ischiocavernous and bulbospongious muscle in the wide pelvic outlet of females (Fig. 5A′), it probably is more effective in the much narrower pelvic outlet of males (Fig. 5B′).

#### *Pelvic venous plexus*

The very well developed valveless venous plexus between the pelvic organs medially and LAM laterally (Batson, 1940) may play an interesting role in urinary continence. A brief increase in pressure on the uterus makes it “sink” a couple of centimeters downward. This feature was long interpreted as a sign of weakened pelvic support, but is now considered physiological (Smith et al., 2013). We interpret this finding as emptying of the venous plexuses. This mechanism may neutralize brief pressure pulses due to, for example, coughing and thus antagonize stress‐induced urinary incontinence. Such a function would explain the presence of these venous plexuses in both females and males, and would become less effective if the pelvic connective tissue becomes more rigid with advancing age.

## CODA

We have started to implement our interactive 3D‐PDF models in our teaching courses at Maastricht University. Many medical students appreciate the opportunity to find out about and comprehend the three‐dimensional construction of the pelvic floor. However, a non‐negligible fraction of students experiences problems in appreciating such complex 3D spatial relationships (see e.g., (Preece et al., 2013)). A major advantage of the sectional approach is, therefore, that 3D‐printing techniques can be used to convert the virtual 3D images into corresponding physical 3D models.

## Supporting information


**Supplemental Figure 1 From sections via reconstruction (Amira 3D) to presentation (Cinema 4D).** The individual sections used for reconstruction are still visible in the Amira 3D reconstruction (A), and were removed by Cinema 4D remodeling (C). In panel B, both Amira and Cinema models are visible to judge accuracy of the remodeling procedure.

**Supplemental Figure 2 Interactive 3D rendering of the topographic anatomy of the female pelvic floor.** The reconstruction is based on 47 structures identified in the CVH5 specimen. After opening the PDF‐file, the 3D‐PDF becomes activated by “clicking” with the mouse on the image. A toolbar appears on the screen that includes the option “model tree”. The model tree displays a material list of all reconstructed structures in the upper box. The list of visible structures can be modified by marking or unmarking a structure. Bilateral structures are present in the list as 2 separate structures to allow building a hemi‐pelvis. A structure can be rendered transparent by selecting that option from the drop‐down menu after selecting the structure with the right mouse button. To manipulate the 3D reconstruction, press the left mouse button to rotate it, the scroll button to zoom in or out, and the left and right mouse buttons simultaneously to move the image across the screen. Note that the 3D‐PDF can be opened on any computer as long as it contains Adobe PDF reader (version 9.3 or higher). Please be advised that a structure can be identified by marking and unmarking it in the model tree or by matching its color code with that of the respective structures presented in supplemental Figure 4.

**Supplemental Figure 3 Interactive 3D rendering of the topographic anatomy of the male pelvic floor.** The reconstruction is based on 45 structures identified in the CVH1 specimen. For viewing instructions, see supplemental Figure 2.

**Supplemental Figure 4** Color codes of structures displayed in the (supplemental) Figures.

## References

[ca23508-bib-0001] Abe T , Kearns CF , Fukunaga T . 2003 Sex differences in whole body skeletal muscle mass measured by magnetic resonance imaging and its distribution in young Japanese adults. Br J Sports Med 37:436–440. 14514537 10.1136/bjsm.37.5.436 PMC1751351

[ca23508-bib-0002] Aigner F , Zbar AP , Ludwikowski B , Kreczy A , Kovacs P , Fritsch H . 2004 The rectogenital septum: morphology, function, and clinical relevance. Dis Colon Rectum 47:131–140. 15043282 10.1007/s10350-003-0031-8

[ca23508-bib-0003] Arakawa T , Hayashi S , Kinugasa Y , Murakami G , Fujimiya M . 2010 Development of the external anal sphincter with special reference to intergender difference: observations of mid‐term fetuses (15‐30 weeks of gestation). Okajimas Folia Anat Jpn 87:49–58. 20882767 10.2535/ofaj.87.49

[ca23508-bib-0004] Baah‐Dwomoh A , McGuire J , Tan T , De Vita R . 2016 Mechanical properties of female reproductive organs and supporting connective tissues: a review of the current state of knowledge. Appl Mech Rev 68:12.

[ca23508-bib-0005] Batson OV . 1940 The function of the vertebral veins and their role in the spread of metastases. Ann Surg 112:138–149. 17857618 10.1097/00000658-194007000-00016 PMC1387927

[ca23508-bib-0006] Beamish JA , He P , Kottke‐Marchant K , Marchant RE . 2010 Molecular regulation of contractile smooth muscle cell phenotype: implications for vascular tissue engineering. Tissue Eng Part B Rev 16:467–491. 20334504 10.1089/ten.teb.2009.0630 PMC2943591

[ca23508-bib-0007] Berglas B , Rubin IC . 1953 Study of the supportive structures of the uterus by levator myography. Surg Gynecol Obstet 97:677–692. 13113550

[ca23508-bib-0008] Béthoux A , Bory S . 1962 Les mécanismes statiques viscéraux pelviens chez la femme à la lumière de l'exploration fonctionnelle du dispositif en position debout. Ann Chir 16:887–916. 13967808

[ca23508-bib-0009] Betschart C , Kim J , Miller JM , Ashton‐Miller JA , DeLancey JO . 2014 Comparison of muscle fiber directions between different levator ani muscle subdivisions: in vivo MRI measurements in women. Int Urogynecol J 25:1263–1268. 24832855 10.1007/s00192-014-2395-9 PMC4140951

[ca23508-bib-0010] Brandao FS , Parente MP , Rocha PA , Saraiva MT , Ramos IM , Natal Jorge RM . 2016 Modeling the contraction of the pelvic floor muscles. Comput Methods Biomech Biomed Engin 19:347–356. 25953072 10.1080/10255842.2015.1028031

[ca23508-bib-0011] Brandon CJ , Lewicky‐Gaupp C , Larson KA , Delancey JO . 2009 Anatomy of the perineal membrane as seen in magnetic resonance images of nulliparous women. Am J Obstet Gynecol 200:e581–e586. 10.1016/j.ajog.2009.03.004 PMC2696929 19375575

[ca23508-bib-0012] Brooks JD , Eggener SE , Chao WM . 2002 Anatomy of the rectourethralis muscle. Eur Urol 41:94–100. 11999473 10.1016/s0302-2838(01)00019-7

[ca23508-bib-0013] Chantereau P , Brieu M , Kammal M , Farthmann J , Gabriel B , Cosson M . 2014 Mechanical properties of pelvic soft tissue of young women and impact of aging. Int Urogynecol J 25:1547–1553. 25007897 10.1007/s00192-014-2439-1

[ca23508-bib-0014] Constantinou CE . 2009 Dynamics of female pelvic floor function using urodynamics, ultrasound and magnetic resonance imaging (MRI). Eur J Obstet Gynecol Reprod Biol 144(Suppl 1):S159–S165. 19303690 10.1016/j.ejogrb.2009.02.021 PMC2691722

[ca23508-bib-0015] Cosson M , Rubod C , Vallet A , Witz JF , Dubois P , Brieu M . 2013 Simulation of normal pelvic mobilities in building an MRI‐validated biomechanical model. Int Urogynecol J 24:105–112. 22707008 10.1007/s00192-012-1842-8

[ca23508-bib-0016] Dai JX , Chung MS , Qu RM , Yuan L , Liu SW , Shin DS . 2012 The visible human projects in Korea and China with improved images and diverse applications. Surg Radiol Anat 34:527–534. 22402591 10.1007/s00276-012-0945-8

[ca23508-bib-0017] De Caro R , Aragona F , Herms A , Guidolin D , Brizzi E , Pagano F . 1998 Morphometric analysis of the fibroadipose tissue of the female pelvis. J Urol 160:707–713. 9720527 10.1016/S0022-5347(01)62764-2

[ca23508-bib-0018] Dorschner W , Biesold M , Schmidt F , Stolzenburg JU . 1999 The dispute about the external sphincter and the urogenital diaphragm. J Urol 162:1942–1945. 10569543 10.1016/S0022-5347(05)68074-3

[ca23508-bib-0019] Fritsch H , Hotzinger H . 1995 Tomographical anatomy of the pelvis, visceral pelvic connective tissue, and its compartments. Clin Anat 8:17–24. 7697508 10.1002/ca.980080103

[ca23508-bib-0020] Fritsch H , Brenner E , Lienemann A , Ludwikowski B . 2002 Anal sphincter complex: reinterpreted morphology and its clinical relevance. Dis Colon Rectum 45:188–194. 11852331 10.1007/s10350-004-6144-x

[ca23508-bib-0021] Frohlich B , Hotzinger H , Fritsch H . 1997 Tomographical anatomy of the pelvis, pelvic floor, and related structures. Clin Anat 10:223–230. 9213037 10.1002/(SICI)1098-2353(1997)10:4<223::AID-CA1>3.0.CO;2-T

[ca23508-bib-0022] Gil‐Vernet JM , Arango O , Álvarez‐Vijande R . 2016 Topographic anatomy and its development in urology in the 20th century. The work of Salvador Gil Vernet. Eur J Anat 20:231–247.

[ca23508-bib-0023] Hikspoors J , Kruepunga N , Mommen GMC , Peeters J , Hulsman CJM , Eleonore Kohler S , Lamers WH . 2019 The development of the dorsal mesentery in human embryos and fetuses. Semin Cell Dev Biol 92:18–26. 10.1016/j.semcdb.2018.08.009 30142441

[ca23508-bib-0024] Jimenez AM , Aguilar JF . 2009 Laparoscopy: learning a new surgical anatomy? Anat Sci Educ 2:81–83. 19363803 10.1002/ase.75

[ca23508-bib-0025] Kathrein A , Klestil T , Birbamer G , Buchberger W , Rabl W , Kuenzel K . 1996 Rotation cryotomy: medical and scientific value of a new serial sectioning procedure. Clin Anat 9:227–231. 8793215 10.1002/(SICI)1098-2353(1996)9:4<227::AID-CA2>3.0.CO;2-B

[ca23508-bib-0026] Kaye KW , Milne N , Creed K , van der Werf B . 1997 The 'urogenital diaphragm’, external urethral sphincter and radical prostatectomy. Aust N Z J Surg 67:40–44. 9033375

[ca23508-bib-0027] Kearney R , Sawhney R , DeLancey JO . 2004 Levator ani muscle anatomy evaluated by origin‐insertion pairs. Obstet Gynecol 104:168–173. 15229017 10.1097/01.AOG.0000128906.61529.6b PMC1415268

[ca23508-bib-0028] Kim JH , Kinugasa Y , Yu HC , Murakami G , Abe S , Cho BH . 2015 Lack of striated muscle fibers in the longitudinal anal muscle of elderly Japanese: a histological study using cadaveric specimens. Int J Colorectal Dis 30:43–49. 25331031 10.1007/s00384-014-2038-0

[ca23508-bib-0029] Kinugasa Y , Arakawa T , Abe S , Ohtsuka A , Suzuki D , Murakami G , Fujimiya M , Sugihara K . 2011 Anatomical reevaluation of the anococcygeal ligament and its surgical relevance. Dis Colon Rectum 54:232–237. 21228674 10.1007/DCR.0b013e318202388f

[ca23508-bib-0030] Kraima AC , West NP , Treanor D , Magee DR , Rutten HJ , Quirke P , DeRuiter MC , van de Velde CJ . 2015 Whole mount microscopic sections reveal that Denonvilliers’ fascia is one entity and adherent to the mesorectal fascia; implications for the anterior plane in total mesorectal excision? Eur J Surg Oncol 41:738–745. 25892592 10.1016/j.ejso.2015.03.224

[ca23508-bib-0031] Lien KC , Mooney B , DeLancey JO , Ashton‐Miller JA . 2004 Levator ani muscle stretch induced by simulated vaginal birth. Obstet Gynecol 103:31–40. 14704241 10.1097/01.AOG.0000109207.22354.65 PMC1226707

[ca23508-bib-0032] Macchi V , Porzionato A , Stecco C , Vigato E , Parenti A , De Caro R . 2008 Histo‐topographic study of the longitudinal anal muscle. Clin Anat 21:447–452. 18561297 10.1002/ca.20633

[ca23508-bib-0033] Muro S , Tsukada Y , Harada M , Ito M , Akita K . 2018 Spatial distribution of smooth muscle tissue in the male pelvic floor with special reference to the lateral extent of the rectourethralis muscle: application to prostatectomy and proctectomy. Clin Anat 31:1167–1176. 30113089 10.1002/ca.23254

[ca23508-bib-0034] Muro S , Tsukada Y , Harada M , Ito M , Akita K . 2019 Anatomy of the smooth muscle structure in the female anorectal anterior wall: convergence and anterior extension of the internal anal sphincter and longitudinal muscle. Colorectal Dis 21:472–480. 30614646 10.1111/codi.14549 PMC6850065

[ca23508-bib-0035] Nakajima Y , Muro S , Nasu H , Harada M , Yamaguchi K , Akita K . 2017 Morphology of the region anterior to the anal canal in males: visualization of the anterior bundle of the longitudinal muscle by transanal ultrasonography. Surg Radiol Anat 39:967–973. 28247085 10.1007/s00276-017-1832-0

[ca23508-bib-0036] Oelrich TM . 1980 The urethral sphincter muscle in the male. Am J Anat 158:229–246. 7416058 10.1002/aja.1001580211

[ca23508-bib-0037] Oelrich TM . 1983 The striated urogenital sphincter muscle in the female. Anat Rec 205:223–232. 6846873 10.1002/ar.1092050213

[ca23508-bib-0038] Oh C , Kark AE . 1973 Anatomy of the perineal body. Dis Colon Rectum 16:444–454. 4769218 10.1007/BF02588867

[ca23508-bib-0039] Pendergrass PB , Reeves CA , Belovicz MW , Molter DJ , White JH . 2000 Comparison of vaginal shapes in Afro‐American, caucasian and hispanic women as seen with vinyl polysiloxane casting. Gynecol Obstet Invest 50:54–59. 10895030 10.1159/000010281

[ca23508-bib-0040] Peng Y , Khavari R , Nakib NA , Boone TB , Zhang Y . 2016 Assessment of urethral support using MRI‐derived computational modeling of the female pelvis. Int Urogynecol J 27:205–212. 26224383 10.1007/s00192-015-2804-8 PMC5519823

[ca23508-bib-0041] Preece D , Williams SB , Lam R , Weller R . 2013 "Let's get physical": advantages of a physical model over 3D computer models and textbooks in learning imaging anatomy. Anat Sci Educ 6:216–224. 23349117 10.1002/ase.1345

[ca23508-bib-0042] Range RL , Woodburne RT . 1964 The gross and microscopic anatomy of the transverse cervical ligament. Am J Obstet Gynecol 90:460–467. 14217646 10.1016/0002-9378(64)90802-6

[ca23508-bib-0043] Sebe P , Oswald J , Fritsch H , Aigner F , Bartsch G , Radmayr C . 2005 An embryological study of fetal development of the rectourethralis muscle: does it really exist? J Urol 173:583–586. 15643263 10.1097/01.ju.0000151248.37875.24

[ca23508-bib-0044] Seow CY , Solway J . 2011 Mechanical and structural plasticity. Compr Physiol 1:283–293. 23737173 10.1002/cphy.c100024

[ca23508-bib-0045] Sergovich A , Johnson M , Wilson TD . 2010 Explorable three‐dimensional digital model of the female pelvis, pelvic contents, and perineum for anatomical education. Anat Sci Educ 3:127–133. 20166225 10.1002/ase.135

[ca23508-bib-0046] Shafik A . 1999 Levator ani muscle: new physioanatomical aspects and role in the micturition mechanism. World J Urol 17:266–273. 10552142 10.1007/s003450050144

[ca23508-bib-0047] Shin DS , Jang HG , Hwang SB , Har DH , Moon YL , Chung MS . 2013 Two‐dimensional sectioned images and three‐dimensional surface models for learning the anatomy of the female pelvis. Anat Sci Educ 6:316–323. 23463707 10.1002/ase.1342

[ca23508-bib-0048] Smith GE . 1908 Studies in the anatomy of the pelvis, with special reference to the fasciae and visceral supports: part II. J Anat Physiol 42:252–270. 17232770 PMC1289162

[ca23508-bib-0049] Smith TM , Luo J , Hsu Y , Ashton‐Miller J , Delancey JO . 2013 A novel technique to measure in vivo uterine suspensory ligament stiffness. Am J Obst Gynecol 209:e481–e487. 23747493 10.1016/j.ajog.2013.06.003 PMC3825841

[ca23508-bib-0050] Spitzer V , Ackerman MJ , Scherzinger AL , Whitlock D . 1996 The visible human male: a technical report. J Am Med Inform Assoc 3:118–130. 8653448 10.1136/jamia.1996.96236280 PMC116294

[ca23508-bib-0051] Stein TA , DeLancey JO . 2008 Structure of the perineal membrane in females: gross and microscopic anatomy. Obstet Gynecol 111:686–693. 18310372 10.1097/AOG.0b013e318163a9a5 PMC2775042

[ca23508-bib-0052] Terminol FCA . 1998 International Anatomical Terminology. Stuttgart: Thieme.

[ca23508-bib-0053] Tobin CE , Benjamin JA . 1945 Anatomical and surgical restudy of Denonvilliers’ fascia. Surg Gynecol Obstet 80:373–388.

[ca23508-bib-0054] Toldt C . 1893 Über die Geschichte der Mesenterien. Verh Anat Ges 8:12–40.

[ca23508-bib-0055] Tsukada Y , Ito M , Watanabe K , Yamaguchi K , Kojima M , Hayashi R , Akita K , Saito N . 2016 Topographic anatomy of the anal sphincter complex and levator ani muscle as it relates to intersphincteric resection for very low rectal disease. Dis Colon Rectum 59:426–433. 27050605 10.1097/DCR.0000000000000565

[ca23508-bib-0056] Umek WH , Morgan DM , Ashton‐Miller JA , DeLancey JO . 2004 Quantitative analysis of uterosacral ligament origin and insertion points by magnetic resonance imaging. Obstet Gynecol 103:447–451. 14990404 10.1097/01.AOG.0000113104.22887.cd PMC1226709

[ca23508-bib-0057] Wallner C , Dabhoiwala NF , DeRuiter MC , Lamers WH . 2009 The anatomical components of urinary continence. Eur Urol 55:932–943. 18755535 10.1016/j.eururo.2008.08.032

[ca23508-bib-0058] Wang Z , Heo M , Lee RC , Kotler DP , Withers RT , Heymsfield SB . 2001 Muscularity in adult humans: proportion of adipose tissue‐free body mass as skeletal muscle. Am J Hum Biol 13:612–619. 11505469 10.1002/ajhb.1099

[ca23508-bib-0059] Wu Y , Dabhoiwala NF , Hagoort J , Shan JL , Tan LW , Fang BJ , Zhang SX , Lamers WH . 2015 3D topography of the young adult anal sphincter complex reconstructed from undeformed serial anatomical sections. PloS One 10:e0132226. 10.1371/journal.pone.0132226 PMC4549266 26305117

[ca23508-bib-0060] Wu Y , Dabhoiwala NF , Hagoort J , Tan LW , Zhang SX , Lamers WH . 2017 Architectural differences in the anterior and middle compartments of the pelvic floor of young‐adult and postmenopausal females. J Anat 230:651–663. 28299781 10.1111/joa.12598 PMC5382597

[ca23508-bib-0061] Wu Y , Dabhoiwala NF , Hagoort J , Tan L‐W , Fang B‐J , Zhang S‐X , Lamers WH . 2018 Architecture of structures in the urogenital triangle of young adult males; comparison with females. J Anat 233:447–459. 30051458 10.1111/joa.12864 PMC6131961

[ca23508-bib-0062] Zhai LD , Liu J , Li YS , Ma QT , Yin P . 2011 The male rectourethralis and deep transverse perineal muscles and their relationship to adjacent structures examined with successive slices of celloidin‐embedded pelvic viscera. Eur Urol 59:415–421. 21144644 10.1016/j.eururo.2010.11.030

[ca23508-bib-0063] Zifan A , Reisert M , Sinha S , Ledgerwood‐Lee M , Cory E , Sah R , Mittal RK . 2018 Connectivity of the superficial muscles of the human perineum: a diffusion tensor imaging‐based global tractography study. Sci Rep 8:17867. 30552351 10.1038/s41598-018-36099-4 PMC6294750

