# Supplementary figures and images for: Interactive three‐dimensional teaching models of the female and male pelvic floor
Source: Clin Anat. 2019 Nov 19;33(2):275–85. doi: 10.1002/ca.23508 (PMC7027585; doi:10.1002/ca.23508)

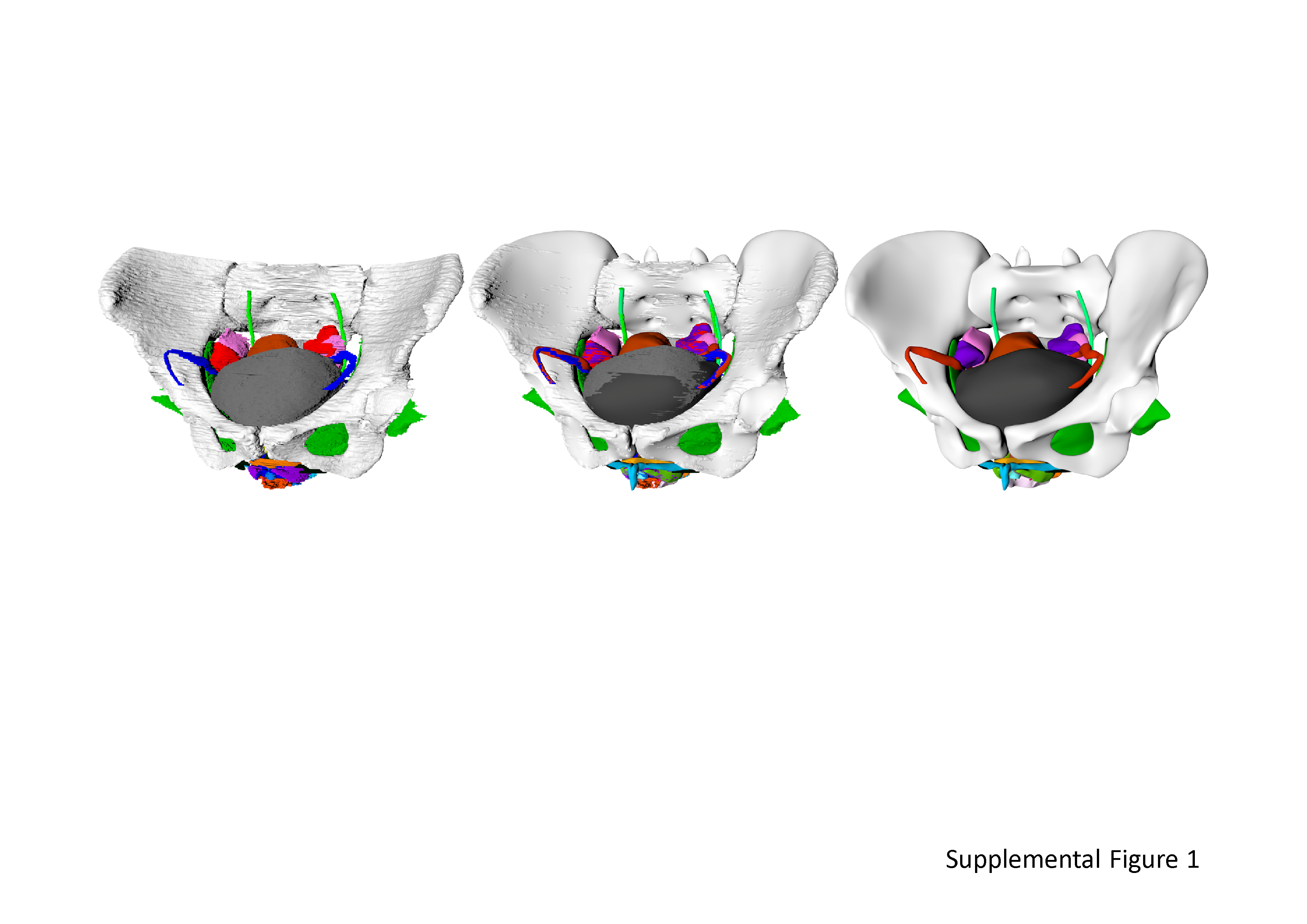

Supplement: Supplementary file 1 — Supplemental Figure 1 From sections via reconstruction (Amira 3D) to presentation (Cinema 4D). The individual sections used for reconstruction are still visible in the Amira 3D reconstruction (A), and were removed by Cinema 4D remodeling (C). In panel B, both Amira and Cinema models are visible to judge accuracy of the remodeling procedure. [file CA-33-275-s001.tif]

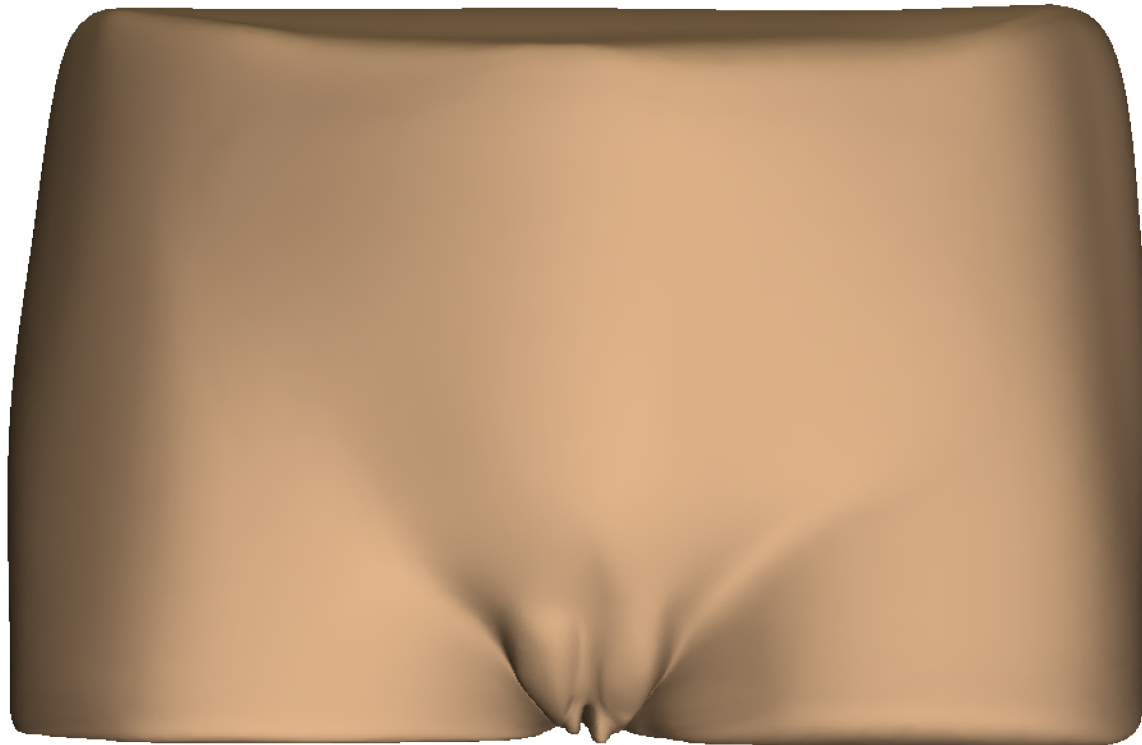

Supplement: Supplementary file 2 — Supplemental Figure 2 Interactive 3D rendering of the topographic anatomy of the female pelvic floor. The reconstruction is based on 47 structures identified in the CVH5 specimen. After opening the PDF‐file, the 3D‐PDF becomes activated by “clicking” with the mouse on the image. A toolbar appears on the screen that includes the option “model tree”. The model tree displays a material list of all reconstructed structures in the upper box. The list of visible structures can be modified by marking or unmarking a structure. Bilateral structures are present in the list as 2 separate structures to allow building a hemi‐pelvis. A structure can be rendered transparent by selecting that option from the drop‐down menu after selecting the structure with the right mouse button. To manipulate the 3D reconstruction, press the left mouse button to rotate it, the scroll button to zoom in or out, and the left and right mouse buttons simultaneously to move the image across the screen. Note that the 3D‐PDF can be opened on any computer as long as it contains Adobe PDF reader (version 9.3 or higher). Please be advised that a structure can be identified by marking and unmarking it in the model tree or by matching its color code with that of the respective structures presented in supplemental Figure 4. [file CA-33-275-s002.pdf]

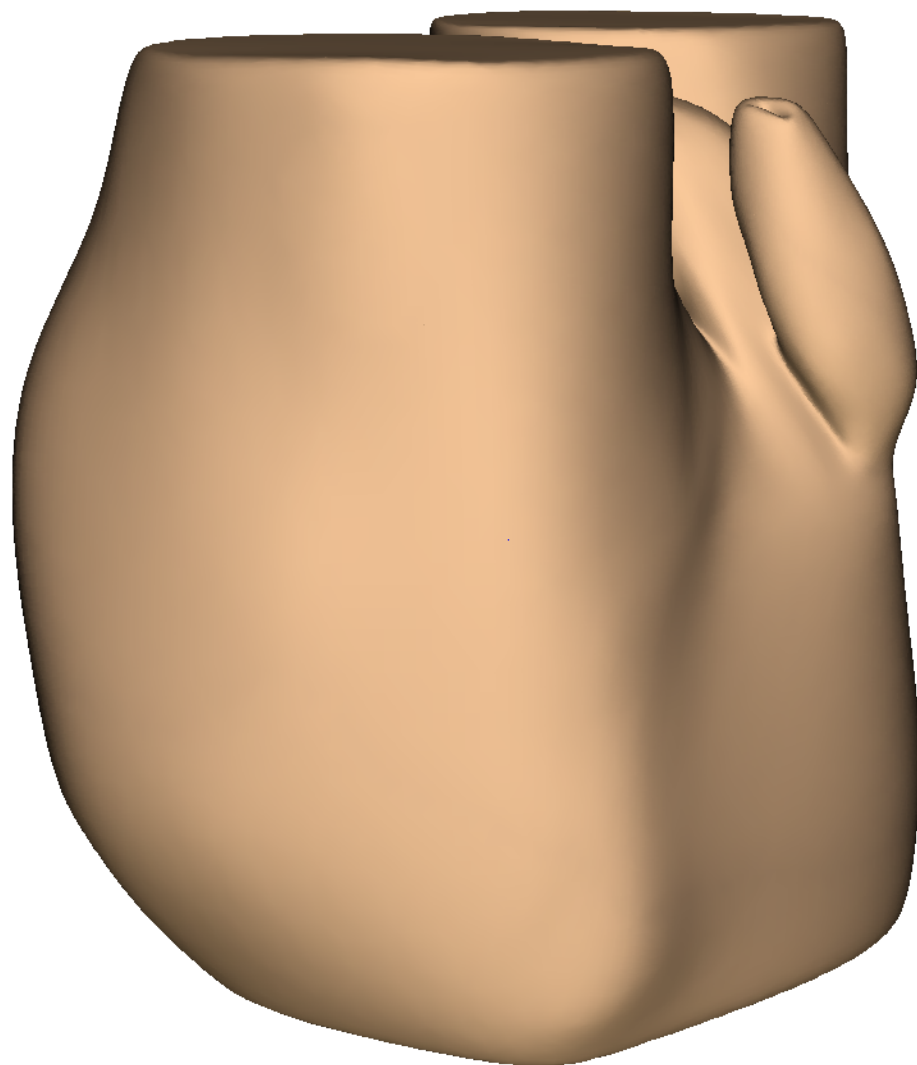

Supplement: Supplementary file 3 — Supplemental Figure 3 Interactive 3D rendering of the topographic anatomy of the male pelvic floor. The reconstruction is based on 45 structures identified in the CVH1 specimen. For viewing instructions, see supplemental Figure 2. [file CA-33-275-s003.pdf]
